# Investigating lightweight and interpretable machine learning models for efficient and explainable stress detection

**DOI:** 10.3389/fdgth.2025.1523381

**Published:** 2025-08-13

**Authors:** Debasish Ghose, Ayan Chatterjee, Indika A. M. Balapuwaduge, Yuan Lin, Soumya P. Dash

**Affiliations:** ^1^ School of Economics, Innovation, and Technology, Kristiania University College, Bergen, Norway; ^2^ Department of Digital Technology, NILU, Kjeller, Norway; ^3^ Department of Information and Communication Technology, University of Agder (UiA), Grimstad, Norway; ^4^ School of Electrical Sciences, Indian Institute of Technology Bhubaneswar, Khordha, Odisha, India

**Keywords:** stress detection, ML models, IoT device, explainable AI, health

## Abstract

Stress is a common human reaction to demanding circumstances, and prolonged and excessive stress can have detrimental effects on both mental and physical health. Heart rate variability (HRV) is widely used as a measure of stress due to its ability to capture variations in the time intervals between heartbeats. However, achieving high accuracy in stress detection through machine learning (ML), using a reduced set of statistical features extracted from HRV, remains a significant challenge. In this study, we aim to address these challenges by proposing lightweight ML models that can effectively detect stress using minimal HRV features and are computationally efficient enough for IoT deployment. We have developed ML models incorporating efficient feature selection techniques and hyper-parameter tuning. The publicly available SWELL-KW dataset has been utilized for evaluating the performance of our models. Our results demonstrate that lightweight models such as k-NN and Decision Tree can achieve competitive accuracy while ensuring lower computational demands, making them ideal for real-time applications. Promisingly, among the developed models, the k-nearest neighbors (k-NN) algorithm has emerged as the best-performing model, achieving an accuracy score of 99.3% using only three selected features. To confirm real-world deployability, we benchmarked the best model on an 8 GB NVIDIA Jetson Orin Nano edge device, where it retained 99.26% accuracy and completed training in 31 s. Furthermore, our study has incorporated local interpretable model-agnostic explanations to provide comprehensive insights into the predictions made by the k-NN-based architecture.

## 1 Introduction

Stress is our body’s response to pressure from challenging situations or life events. It can manifest as a feeling of being overwhelmed or under pressure. While some amount of stress can be beneficial, experiencing overwhelming stress over an extended period is often referred to as chronic or long-term stress, which requires attention. Chronic stress not only affects emotional well-being but also increases susceptibility to severe health issues such as cardiovascular disease and cognitive impairment. Long-term or repeated stress can have harmful effects on both sleep and memory (1). Furthermore, it can lead to changes in eating habits, increased alcohol consumption, and smoking. Additionally, chronic stress can negatively impact the immune system, increasing the risk of depression (2). Psychosocial stress, which is caused by specific social situations, is also believed to be a cause of depression, stroke, heart attack, and cardiac arrest (3, 4). In modern work environments, particularly in high-stress professions, early detection of stress is crucial for mitigating its long-term effects, emphasizing the need for robust and interpretable models that can function in real-time settings. Therefore, there has been a significant study over the recent years by researchers towards investigating various detection and analysis methods for abnormal stress in the human body.

Most of the available research studies for stress detection have indicated the potential to identify stress through vital sign data. Although numerous studies have focused on HRV-based stress detection, challenges remain in identifying the minimal feature sets that maintain high accuracy while reducing computational load. This becomes particularly relevant for edge computing and wearable technology applications. Vital signs are measurements of several physiological functions, including heart rate (HR), blood pressure, respiration rate (RR), and body temperature, that can offer valuable insights into an individual’s overall health and well-being. According to (5), when under stress, the HR, RR, and systolic blood pressure (SBP) are the most affected vital signs. Moreover, heart rate variability (HRV) is a prevalent indicator of a person’s stress level since it can reflect the balance between the sympathetic and parasympathetic nervous systems (6). Moreover, the studies in (7) have highlighted that stress can manifest in various ways, such as decreased HRV and changed respiratory patterns. Therefore, in this study, HRV is considered as a means of determining stress levels. Therefore, this work is motivated by the *hypothesis* that a limited subset of HRV features, when processed by efficient Machine learning (ML) models, can serve as reliable stress biomarkers with minimal resource consumption.

HRV represents the variance in time between every heartbeat called inter-beat intervals (IBIs) (8). HRV has been increasingly recognized as a useful measure of autonomic nervous system (ANS) activity and a potential indicator of stress levels and related mental health conditions. Research has shown that a reduction in HRV is associated with various psychiatric disorders, including depression, anxiety, post-traumatic stress disorder, and schizophrenia (9). A reduced HRV condition indicates a higher likelihood of current or future health issues as the body becomes less adaptable and experiences difficulty coping with changing circumstances. Consequently, HRV typically increases when the heart rate is slow and decreases when it accelerates, such as during exercise or periods of stress.

The growing accessibility of wearable devices capable of tracking HRV, coupled with advancements in ML, creates new opportunities for stress monitoring in everyday environments. This technological evolution has made HRV monitoring more accessible and convenient, enabling remote monitoring in various settings. However, there is a gap in the literature regarding lightweight ML models that can achieve high accuracy while remaining feasible for deployment on IoT edge devices. This gap presents a key opportunity for the development of stress detection models optimized for real-time, low-resource environments.

Machine learning models are now commonly deployed on edge IoT devices for detection and classification tasks and can play a vital role in stress detection, enabling real-time analysis and decision-making. In the healthcare domain, it is essential for ML models to be interpretable and explainable. Fortunately, the field of explainable artificial intelligence (AI) has evolved, offering methods like local interpretable model-agnostic explanations (LIME) (10) and Shapley additive explanations (SHAP) (11). These methods focus on enhancing our understanding and interpretation of the behavior of AI systems, ensuring transparency in the decision-making process. In this work, we integrate such explainability techniques to ensure that our stress detection models are not only accurate but also transparent, fostering trust in their predictions.

In this study, our primary objective is to evaluate the effectiveness of lightweight machine learning models for stress detection, given that advanced or deep learning models are computationally expensive. These lightweight models are designed to be efficient, accurate, and interpretable, making them suitable for real-time deployment on IoT edge devices. By focusing on such models, we aim to address the practical constraints of resource-limited environments while providing interpretable and reliable stress detection. To the best of our knowledge, this is one of the first studies to present AI-assisted stress detection using edge-based IoT devices. Furthermore, it incorporates explainable AI techniques, such as LIME, to enhance the interpretability and transparency of the AI-driven decision-making process. The contributions of this study are as follows:

• We perform a rigorous evaluation of conventional ML algorithms, with an emphasis on feature selection and hyperparameter tuning. Using ANOVA for feature selection and grid search for tuning, we optimize model performance, with k-NN achieving a notable 99.3% accuracy using only three features.
• We compare our models with state-of-the-art methods, showing that our approach achieves similar or better accuracy with reduced computational demands.
• To enhance interpretability, we integrate LIME into our modeling framework, which clarifies the decision-making process of our models.
• We conduct a quantitative analysis to evaluate model efficiency and cross-validate its accuracy using both simulations and real-life IoT edge devices.

The structure of this paper is the following: Section 2 describes the related work. In Section 3, the conceptual framework for multi-class stress classification is presented. Techniques and procedures employed in the study are explained in Section 4, including different lightweight ML methods. Further, performance metrics are presented in Section 5, while Section 6 outlines the experimental methodology and illustrates and discusses the obtained experimental results. Section 7 highlights the limitations. Finally, Section 8 concludes this paper.

## 2 Related work

In this section, we review the recently proposed ML models that have been used for predicting patients’ health conditions based on vital sign data and highlight their significant findings. Current research studies utilize ML algorithms to extract patterns and relationships from large sets of vital sign data, such as HRV, that might indicate an abnormal situation, providing a powerful tool for identifying meaningful predictors and improving diagnostic accuracy.

### 2.1 Machine learning for stress detection

In order to predict the patient’s stress conditions using vital sign data, different ML techniques have been tested. Real-time monitoring of the patient’s vital signs allows for timely predictions and alerts if the predicted condition deviates from the normal range. The process involves several steps, including data preprocessing, feature extraction, model training, and prediction.

The article (12) introduces a framework for early identification of mental stress levels, named MuLHiTA, which utilizes a multi-branch long short-term memory (LSTM) and hierarchical temporal attention for multi-class classification. The performance of MuLHiTA is evaluated on three selected datasets, and the average classification accuracy is reported. A bidirectional LSTM (BLSTM) network was adopted in (12) since it could extract more temporal features than LSTM. In (13), a deep neural network (DNN) model that uses image encoding for classifying an individual’s mental stress is proposed. The study collected data considering various aspects, such as ECG signals, computer logging, facial expressions, body postures, and skin conductance, which were then converted into data frames. However, the adopted ML models in (12, 13) cannot be considered as lightweight.

In (14), an improved unobtrusive ML-based method for remote mental stress detection is proposed by using two continuous wave radars. A CatBoost Classifier is used to classify between relaxed and stress states. Results reveal that the proposed method is capable of detecting mental stress with an accuracy of 89%. However, the data was obtained from young, practically healthy persons only. In (15), an estimation of the left ventricle ejection fraction (LVEF) levels of coronary artery disease (CAD) patients is proposed by using hourly HRV data. Therein, support vector regression (SVR) models were applied to estimate LVEF from ECG-derived HRV data. A per-hour step-wise feature selection approach is proposed to ensure that all possible feature combinations are included in order to fine-tune the model at every hourly interval and reduce the estimation errors. However, the classification is limited to binary classes in both (14, 15), which may not be adequate to identify the exact health condition of a patient.

Furthermore, employing deep learning, an adaptive single and multiple modality data compression scheme is proposed in (16) targeting mobile health applications (mHealth). In that work, an energy and resource-aware framework has been considered for medical data delivery. Their proposed optimization framework illustrates its efficiency in reducing the total energy consumption of the system. In addition to the studies mentioned, several other studies in the literature have focused on binary/multilevel stress detection using AI algorithms, including (17–24). However, it is worth noting that none of these studies have incorporated an explainable AI framework to interpret their developed models.

When working with larger datasets, the time needed for data collection can affect the accuracy of classification results. Additionally, it is important to consider that the effectiveness of ML approaches used on patient health data is strongly influenced by the quality of the data and the selected features. As the number of features necessary to achieve the desired accuracy increases, the system becomes more complex and expensive. Therefore, in this study, we focus on developing a low-cost and highly accurate ML model to predict stress levels based on HRV data. We believe that this approach has the potential to improve the accuracy and speed of stress prediction while reducing costs and increasing applicability for practical scenarios. On the other hand, most of the existing machine learning models, for instance, deep learning algorithms, are known for their black-box nature, making it difficult to explain the underlying reasons behind the predictions. Nevertheless, interpretable models (25) are required, especially in mental health applications, to provide insights into the factors contributing to stress. Clinicians can accurately identify mental health conditions in different situations when they possess knowledge about the underlying factors contributing to the prediction. Furthermore, the utilization of explainable AI systems in patient diagnosis can help to build trust between doctors and the system. This is because doctors can comprehend the specific methods and reasoning employed by the AI system to arrive at a diagnosis. Therefore, in this work, we incorporate interpretable model-agnostic explanations as well.

In Table 1, we provide a qualitative comparison between our study and other state-of-the-art studies in the field of stress detection. The comparison presented in Table 1 highlights the distinctive aspects of our study in stress detection, setting it apart from other studies. Firstly, we leverage a diverse range of lightweight models, such as k-NN, Decision Tree, Logistic Regression, and Gaussian Naive Bayes. These models are renowned for their simplicity, efficiency, and effectiveness, allowing us to strike a balance between accuracy and computational efficiency. Secondly, we conduct a comprehensive complexity analysis, considering crucial factors like execution time and time complexity. By carefully assessing these aspects, we ensure that our models are efficient and capable of providing real-time stress detection capabilities. Thirdly, we prioritize the incorporation of explainable AI by utilizing the LIME method, which grants us valuable insights into the decision-making process of stress classification. These unique features collectively contribute to the interpretability, efficiency, and accuracy of our stress detection approach, distinguishing it from other studies in the field.

**Table 1:** Qualitative comparison with other state-of-the-art studies.

Ref.	Dataset	Explainable AI	Complexity analysis	Lightweight models
Xia et al. (12)	MIST, STEW, DMAT	N.A.	run-time	No (LSTM)
Ghosh et al. (13)	WESAD, SWELL-KW	N.A.	N.A	No (DNN)
Anishchenko and Turetzkaya (14)	From experiment	N.A.	N.A	Yes (CatBoost -based on decision trees)
Ren et al. (26)	Created dataset based on actual medical records	Bayesian Network	N.A	No (CNN)
Alkhodari et al. (15)	IDEAL	N.A.	Per-hour feature selection	Yes (SVR)
Sriramprakash et al. (23)	SWELL-KW	N.A.	N.A	Yes (SVM)
Sarkar and Etemad (22)	SWELL-KW	N.A.	N.A	No. CNN
Koldijk et al. (19)	SWELL-KW	N.A.	N.A	Yes (SVM)
Albaladejo-González et al. (17)	SWELL-KW	N.A.	N.A	Yes (MLP)
Perangin-Angin and Bachtiar (21)	SWELL-KW	N.A.	N.A	No (ELM/ANN)
Khan et al. (18)	SWELL-KW, DREAMER	N.A.	Execution time	Yes (extra tree)
Mortensen et al. (20)	SWELL-KW	N.A.	Execution time	No (1D-CNN)
This study	SWELL-KW	LIME	Execution time and time complexity	Yes (k-NN, decision tree, logistic regression, and Gaussian Naive Bayes)

## 3 Framework for stress classification

**Figure 1:**
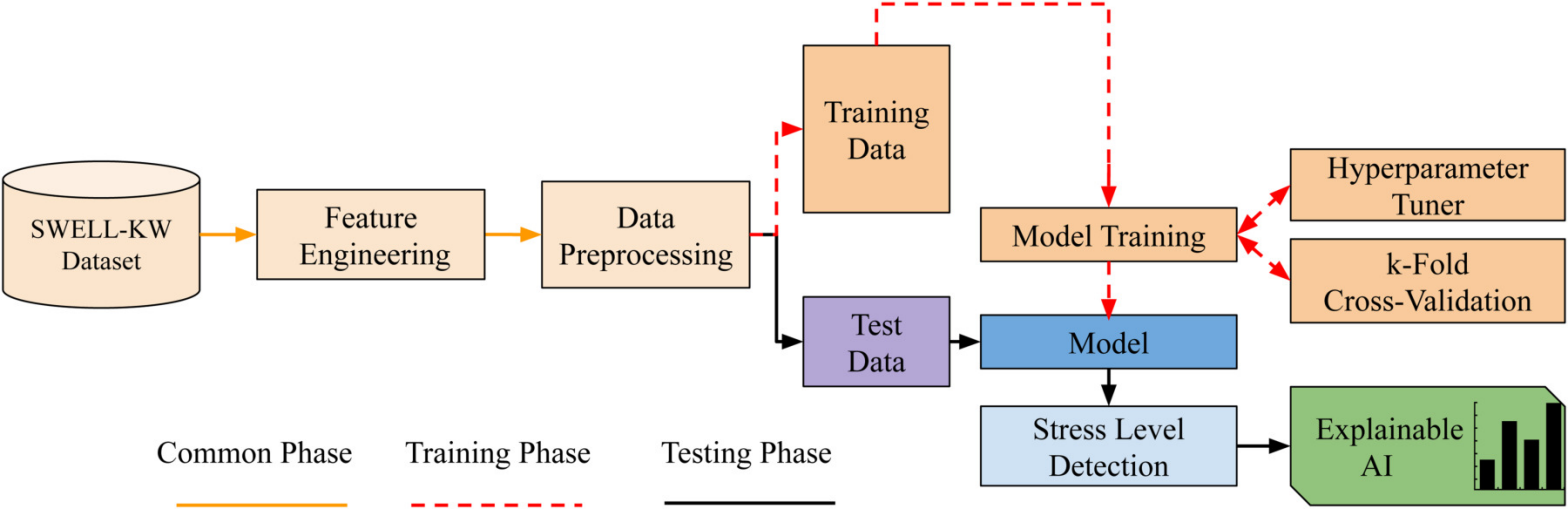
Conceptual framework of the proposed stress level classification.

This section outlines the framework for multi-class stress classification and presents an overview of the entire process. The framework is depicted below in a series of concise points, and illustrated in Figure 1.

• Dataset and data preparation: The SWELL-KW (27) dataset is loaded and the necessary data cleaning procedures are performed.
• Feature engineering: A feature selection method is applied to rank the features based on their scores and the top features are selected.
• Data preprocessing: The target variables are encoded for categorical data, and the features are normalized for consistent scaling.
• Data partitioning: The dataset is partitioned into separate subsets for training and testing. The training dataset is used to train the model, while the testing dataset is used for validation purposes to evaluate the model’s performance.
• Model training: A ML model is constructed, and its hyperparameters are optimized using suitable techniques. The specific hyperparameters to be tuned are specified. To evaluate the performance of the model, k-fold cross-validation is employed.
• Model evaluation: Once the model has been fine-tuned, it is subjected to evaluation on unseen data, specifically the test dataset. This evaluation involves measuring the performance of the model using predefined metrics to gain insights into its effectiveness and accuracy.
• Interpretability of model predictions: Explainable AI is utilized to gain insights into the factors influencing the decisions of the model.

## 4 Methods

In this section, we provide a comprehensive description of the various techniques and procedures employed in the study. This encompasses the dataset used, the feature selection method, the data preprocessing techniques applied, the models adopted, and the hyperparameter tuning and cross-validation approaches utilized. Furthermore, the model training process, the explainable AI method used for interpretability, as well as detailed information about the experimental setup are also explained.

### 4.1 Dataset

We utilize the SWELL-KW dataset, which is collected within the *Smart reasoning for well-being at home and at work (SWELL)* project (28). The SWELL-KW dataset encompasses data captured from diverse sensors, such as computer logging, video, Kinect 3D, and body sensors (19, 27). To prepare the data for analysis, the researchers from the SWELL project perform a series of preprocessing steps. These steps include aggregating computer interactions, extracting facial expressions and postures, capturing heart rates, and measuring skin conductance levels from the collected data. The SWELL-KW dataset consisted of 25 participants who were primarily students with native heritage from the Delft University of Technology in the Netherlands, along with interns from TNO, an independent research organization. Among the participants, 17 were male, while 8 were female, with an average age of 25 years.

The study for generating the dataset took place in a professional setting, where individuals were engaged in common administrative computer tasks that demand a certain level of cognitive exertion. These tasks included web searches, email reading, and presentation creation. The researchers manipulated the working conditions of the participants by introducing two types of stress-inducing factors: interruptions from emails and time constraints. The SWELL-KW dataset includes HRV measurements used for stress and user modeling. More specifically, it contains time-domain and frequency-domain features of HRV. In addition to HRV, the researchers also recorded the subjective experiences of the participants, such as task load, mental effort, mood, and perceived stress. During the study, each participant was exposed to three different working environments, and medical professionals labeled the corresponding data as follows:

• *No stress*: Participants were given the freedom to work on activities as long as they needed, with a maximum duration of 45 minutes. However, they were unaware of the specific time limit for the task.
• *Time pressure*: In this condition, the time allotted to complete the same job was reduced to two-thirds of the normal duration, creating a sense of time pressure.
• *Interruption*: Participants experienced interruptions when they received eight emails while engaged in a given activity. Some emails were relevant to their tasks and required specific actions, while others were completely unrelated to their ongoing tasks.

#### 4.1.1 Exploratory data analysis

**Figure 2:**
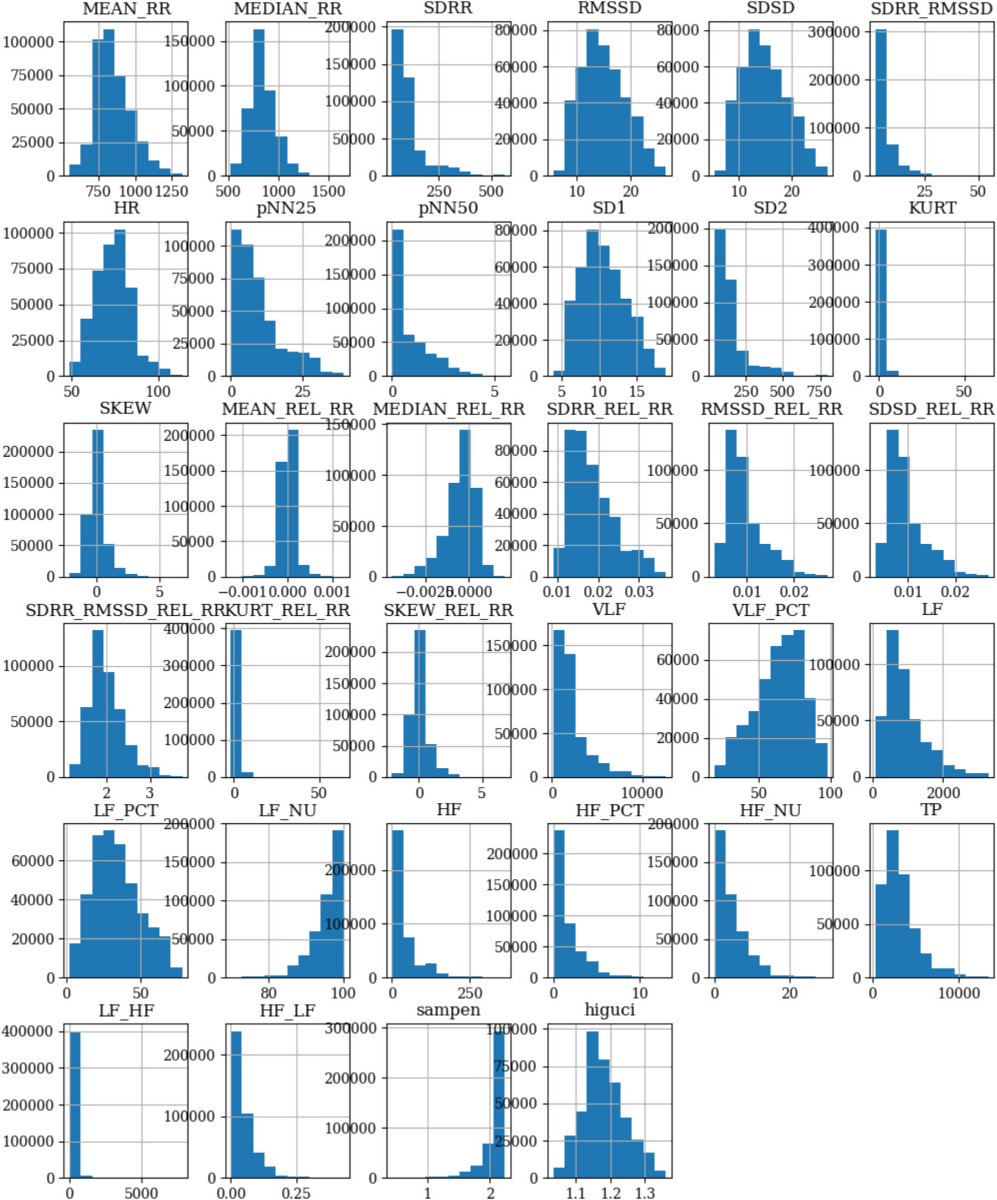
Distribution of numerical variables across different features in the SWELL-KW dataset.

**Figure 3:**
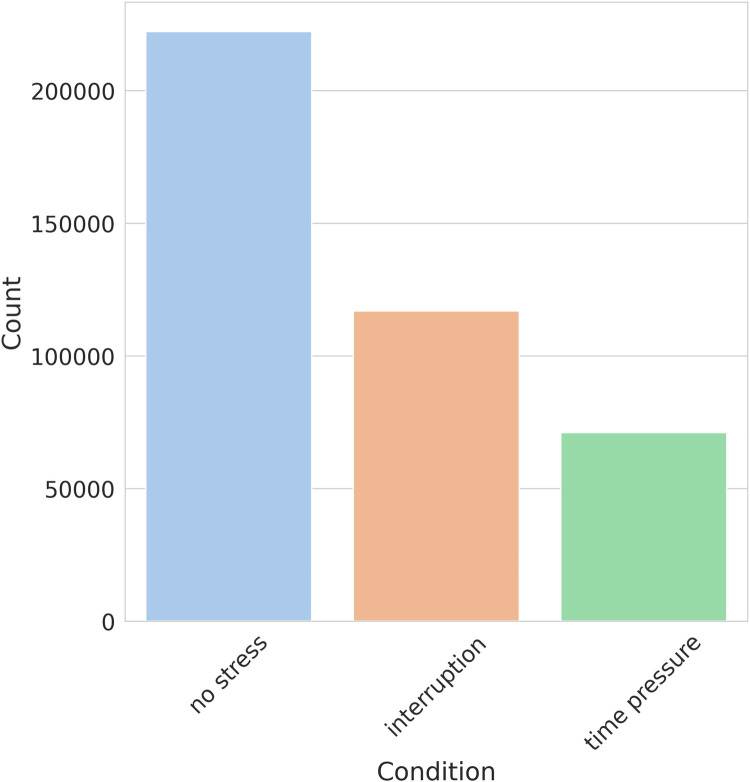
Distribution of stress levels in SWELL-KW.

As part of the exploratory data analysis, we examined the shape of the dataset, revealing that it consists of 410,322 records with 35 columns. These columns encompass 34 HRV features and one column is dedicated to stress labels. The stress labels indicate the different stress conditions associated with each record, namely, *No stress*, *Time pressure*, and *Interruption*. No missing data or null values were found in the dataset. The distribution of numerical variables across different features is visualized using a histogram shown in Figure 2. Furthermore, it is observed from the stress class distribution in Figure 3 that the dataset is imbalanced among the three classes implying that the dataset is not evenly distributed across the stress categories. To maintain consistency with existing literature, the original dataset was kept unchanged for a fair comparison. However, acknowledging the importance of addressing the class imbalance, a balanced dataset is created, and the performance of the developed models is evaluated and presented in Section 7.

**Figure 4:**
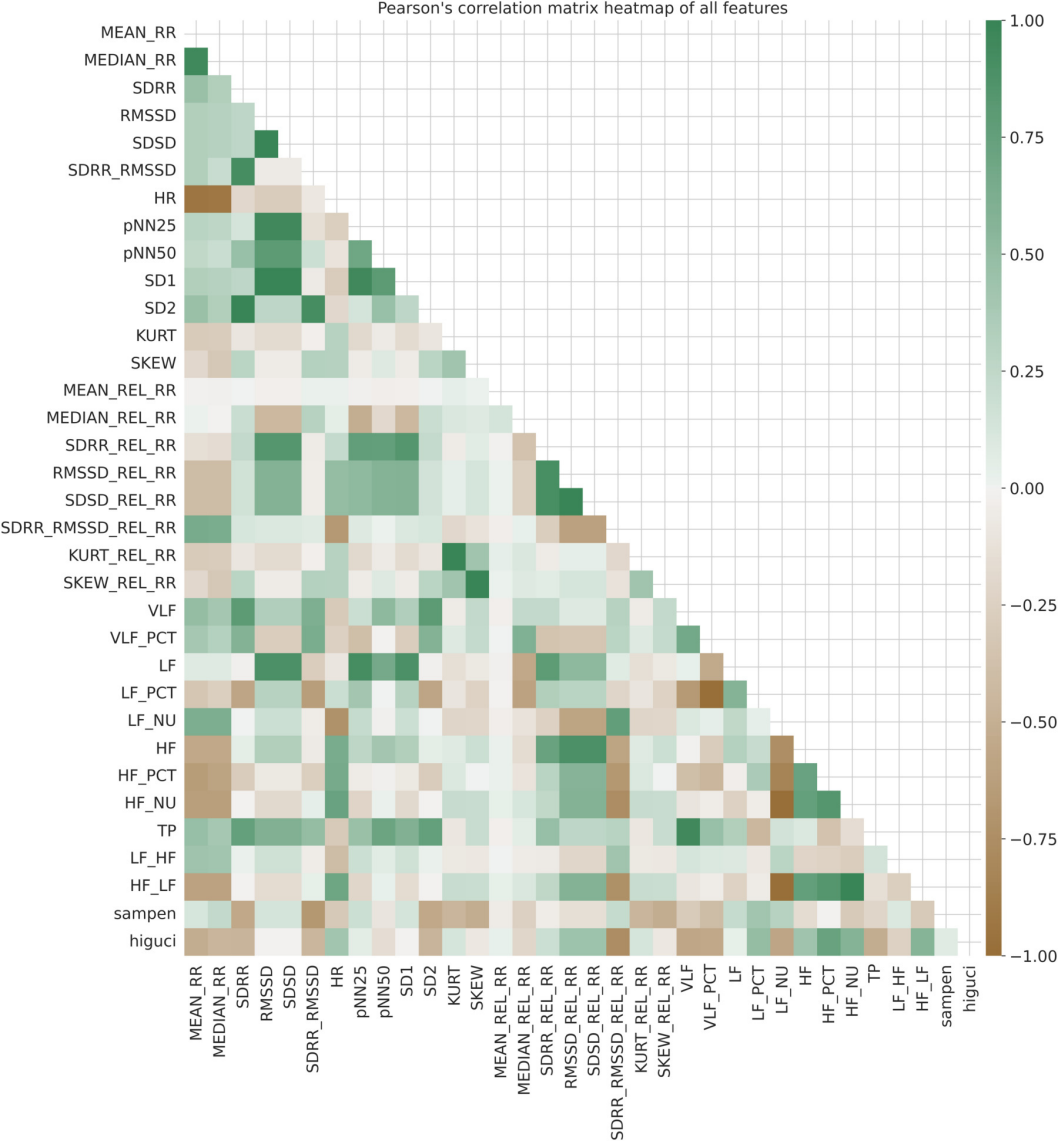
Pearson’s correlation matrix heatmap of all features in SWELL-KW (27) dataset.

Additionally, we utilized Pearson’s correlation matrix, as shown in Figure 4, to identify relationships and patterns between features. The color variations in Figure 4 represent the scale of correlations, ranging from −1 to 1. A value of 1 indicates a strong positive correlation, 0 represents no correlation, and −1 indicates a strong negative correlation. Upon examining Figure 4, it is evident that there are strong correlations among several features. For instance, *SD1* demonstrates strong positive correlations with *SDSD* and *RMSSD* than other features in the correlation matrix. Similar patterns of strong correlations can be observed among other features as well.

The above correlation analysis provided an overview of the strength and direction of associations within the SWELL-KW dataset. Specifically, high correlations (close to 1 or −1) between pairs of features indicate the presence of similar or redundant information. On the other hand, features with weak correlations (close to 0) have limited contributions to the predictive power of ML models. These observations triggered our motivation to assess feature importance within the SWELL-KW dataset using feature selection techniques (29), aiming to eliminate highly correlated features and reduce multicollinearity. Note that, multicollinearity can be defined as the presence of high correlations between two or more independent variables (30).

### 4.2 Feature selection methods

In this study, we employ feature selection methods to analyze the importance of different features in the SWELL-KW dataset. We utilize two feature selection methods, i.e., ANOVA and extra trees classifier, to cross-validate and compare the results obtained from each method.

#### 4.2.1 Analysis of variance (ANOVA)

ANOVA F-test (31) is a powerful tool for analyzing the relevance of features in a classification task. It conducts a parametric statistical hypothesis test to determine if the means of two or more data samples are derived from the same underlying distribution. Through the computation of the F-statistic, which represents the ratio of variances, ANOVA enables the comparison and evaluation of differences among sample means. Moreover, it provides insights into the proportion of variance that can be attributed to different factors (31).

ANOVA is particularly suitable for statistical analysis when the input data comprises numerical features and the outcome variable is categorical. Due to this fact, given the SWELL-KW dataset, characterized by numerical feature values and a categorical outcome, the utilization of ANOVA is deemed advantageous. By applying the ANOVA method to the SWELL-KW dataset, we can effectively identify and extract the most relevant features, facilitating their subsequent ranking and utilization in the ML model development.

#### 4.2.2 Extra trees classifier

The extra trees classifier, initially introduced by (32), is a tree-based ensemble method specifically designed to address supervised classification and regression problems. Its fundamental principle revolves around the introduction of strong randomization in attribute and cut-point selection during the node-splitting process. By incorporating this randomized approach, the extra trees classifier distinguishes itself from other tree-based methods and exhibits unique characteristics and effectiveness in handling diverse predictive tasks.

The extra trees classifier has gained recognition as an efficient feature selection method, particularly for categorical tasks. It utilizes bagging and random subspace techniques, allowing for the selection of random attribute values for top-down splitting (33). In this study, we apply the extra trees classifier to identify and extract the most relevant features of the SWELL-KW dataset. Additionally, we cross-validate the obtained results on feature importance via the extra tree classifier with those achieved through the ANOVA method.

### 4.3 Hyperparameter tuning

Hyperparameter tuning in machine learning involves the iterative exploration of different configurations to select the optimal combination of hyperparameter values that maximize model performance on a given dataset. In this study, we have adopted *grid search* as the hyperparameter tuner for the proposed lightweight ML models.

Grid search has been a widely used approach for hyperparameter optimization since the 1990s. It is characterized by an exhaustive search through a manually specified subset of the hyperparameter space of a learning algorithm. By systematically exploring different combinations of hyperparameter values, grid search aims to identify the optimal configuration that maximizes the model performance. To guide the grid search algorithm, the defined performance metrics are typically utilized, often measured through cross-validation on the training set (34).

### 4.4 k-fold cross-validation

Cross-validation is a model validation technique used to assess how well the results of a statistical analysis will generalize to independent data sets (35). In this study, we use k-fold cross-validation to guide the grid search process for hyperparameter optimization. k-fold cross-validation is a widely adopted model validation technique, known for its popularity and effectiveness in assessing the generalization capabilities of statistical analyses. It involves an iterative process of partitioning the training data into k subsets or folds. During each iteration, one fold is designated as the test set, while the remaining k−1 folds are used for training the model. Accordingly, the performance of the model is recorded and evaluated. By the end of the process, the average performance across all iterations is computed, providing a reliable estimate of the performance of the model.

### 4.5 Lightweight ML models

Lightweight ML models are known for their low memory and computational requirements, making them suitable for resource-constrained environments such as IoT devices, mobile devices, and embedded systems. In this study, we have chosen k-Nearest Neighbors, Decision Tree, Logistic Regression, and Gaussian Naive Bayes as the ML models for evaluating their performance on the SWELL-KW dataset. These models are specifically selected due to their ability to operate efficiently with limited computational resources (36).

#### 4.5.1 k-nearest neighbors (k-NN)

The k-NN algorithm is a non-parametric supervised ML algorithm that can be used to solve classification and regression problems, which was initially proposed by Evelyn Fix and Joseph Hodges (37). The k-NN algorithm works by finding the k instances from the training dataset that are nearest to a new instance and classifying the new instance as belonging to the most common class among those k neighbors. When employed for classification tasks, the class of a given new instance is determined by the majority vote of its k-nearest neighbors.

Assume $S_{k}(x)$ is the set of k points in the training set closest to a new instance x based on the Euclidean distance metric. Then, the k-NN algorithm can be described by the following pseudo-mathematical function:

$$c(x)=\arg\;\max_{c\in C}\sum_{y\in S_{k}(x)}I(c\equiv c_{y}),$$

where c(x) is the predicted class for the instance x, C is the set of all classes, and $c_{y}$ is the class of instance y in $S_{k}(x)$. Moreover, I(⋅) is an indicator function, which is equal to 1 if the condition inside the parentheses is true, and 0 otherwise, and $\arg\;\max_{c\in C}$ is to choose the class c that maximizes the $\sum_{y\in S_{k}(x)}I(c\equiv c_{y})$.

In the k-NN algorithm, the distance between each data point is calculated to determine the nearest points. The selection of a distance metric is crucial to the measurement of similarity or dissimilarity between instances in the space of features. The methods of measuring distance are—Euclidean distance (it calculates the straight-line distance between two points in space), Manhattan distance or L1 distance (it calculates the total absolute difference between the points’ coordinates), Minkowski distance (it is a generalization of the former two, and it is parameterized by a value “p” that determines the degree of distance calculation. When p=1, it reduces to the Manhattan distance, and when p=2, it becomes the Euclidean distance), the Cosine distance (it calculates the degree to which two vectors are dissimilar, ignoring their magnitude), and the Hamming distance (it calculates the number of positions at which two strings of equal length differ). The selection of a distance metric is based on the nature of the data and the specific goal of the targeted problem. In this research, we’ve utilized the Euclidean distance in k-NN as the dimensions of the space for features are all continuous and have a significant geometric meaning.

#### 4.5.2 Decision tree

The decision tree is another non-parametric supervised ML algorithm that is commonly used for classification tasks (38). It operates by creating a tree structure, where each leaf node denotes a class label and each internal node denotes a feature, and the paths from the root to leaf nodes characterize the classification rules. To construct a decision tree, the algorithm identifies which attributes or features fit the root node and each level, guided by the principle of maximizing information gain (39). For a dataset S, the entropy H(S) of the dataset S is given by

$$H(S)=-\sum_{c\in \mathrm{Classes}}p_{c}\log_{2}\;p_{c},$$

where $p_{c}$ is the fraction of instances in S belonging to class c. Given the dataset S and feature f, the information gain, IG(S,f) is calculated as

$$IG(S,f)=H(S)-\sum_{v\in V(f)}\frac{\left|S_{v}\right|}{\left|S\right|}H(S_{v}),$$

where $S_{v}$ refers to the subset of instances in S in which f has the value v. Note that V(f) is the collection of all potential values for f. Recursively, this process is carried out until either all instances in the subset belong to the same class, or there are no more remaining attributes to the test.

#### 4.5.3 Logistic regression

Logistic regression is a parametric supervised ML algorithm extensively used for binary classification tasks (40). The primary objective of logistic regression is to estimate the likelihood of an instance being associated with a specific class. In the context of multi-class classification, softmax regression is typically used as logistic regression.

Given a data point x, the conditional probability that it belongs to a specific class i is given by

$$p(y=i|x;\theta )=\frac{e^{\theta^{(i)T}x}}{\sum_{\;j=1}^{K}\;e^{\theta^{(j)T}x}}$$

where K is the number of classes, $\theta^{\left(i\right)}$ represents the weights or coefficients for the input x for class i and $\sum_{\;j=1}^{K}\;e^{\theta^{(j)T}x}$ represents the sum of exponential terms for all classes, which serves as a normalizing factor to ensure that the sum of the probabilities of all classes is 1. The class with the highest probability is chosen as the output class.

#### 4.5.4 Gaussian Naive Bayes

Gaussian Naive Bayes is an extension of Naive Bayes and it is a probabilistic ML model commonly used for classification problems. The fundamental expression of Naive Bayes guided by Bayes theorem (41, 42), calculates the probability of each class considering the input features as follows.

$$P(B_{k}|a_{1},\ldots ,a_{n})=\frac{P(B_{k})\cdot P(a_{1},\ldots ,a_{n}|B_{k})}{P(a_{1},\ldots ,a_{n})}$$

where $P(B_{k}|a_{1},\ldots ,a_{n})$ represents the posterior probability of class-given predictors while $P(B_{k})$ signifies the prior probability of the class. $P(a_{1},\ldots ,a_{n}|B_{k})$ denotes the likelihood, which is the probability of predictors given the class, and $P(a_{1},\ldots ,a_{n})$ is the prior probability of the predictors.

Gaussian Naive Bayes is particularly used when the features are continuous and the likelihood of the features is assumed to follow a normal distribution as already mentioned.

### 4.6 Explainable AI

Explainable AI (XAI) is a set of processes and techniques that allows humans to understand the logical reasoning behind decisions or predictions made by AI models. The explanation of the problem in machine learning classification is called the challenge of understanding and interpreting how a classifier makes predictions or decisions. While machine learning models can have high accuracy, they typically function as black boxes, which makes it difficult to understand the underlying principles behind their predictions. There are several methods of dealing with the explanation issue in ML classification: Interpretability Techniques, Local Explanations, Global Explanations, and Model-specific Techniques. Addressing the explanation problem in ML classification helps build trust, understanding, and acceptance of machine learning models. In this study, we have utilized LIME to interpret the results or decisions made by our developed ML models.

#### 4.6.1 LIME

LIME is an XAI method for explaining predictions of ML models, developed by Marco Ribeiro (10). LIME provides local, interpretable explanations by approximating the black-box model’s behavior around a specific instance. It helps understand why a model made a particular prediction for a given instance and provides insights into the features that influenced the prediction. LIME has been designed with two fundamental characteristics. The first is its model-agnostic nature, meaning that it can work with any type of ML model, no matter the complexity or structure. The second characteristic is its focus on locality; LIME’s goal is to elucidate individual, or local, predictions rather than attempting to explain the behavior of the model as a whole.

The process of LIME begins by choosing an ML model, regardless of its type, and a reference point that requires explanation. New data points are then created across the $R^{p}$ space through the sampling of X values from a normal distribution based on the training set. The ML model predicts the Y coordinates for these points, guaranteeing a perfect fit on the model’s decision surface. Weights are assigned to these points based on their closeness to the chosen point, with higher weights attributed to points that are closer. An easily interpretable model, like a Linear Model or Decision Tree, is then trained on this weighted dataset. The equation for this training is given as

$$E(Y)=\beta_{0}+\sum_{\;j}\beta_{j}X_{j}.$$

In this equation, the $\beta_{j}$ coefficients serve as the explanation for LIME, offering insights into the decision-making of the complex ML model in the vicinity of the reference point. LIME uses different color codes to highlight the significance of features. These color codes can be used to represent the importance or contribution of a feature to the LIME explanation to facilitate the interpretation of the results.

## 5 Performance metrics

In the evaluation of the analyzed multi-class stress classification models, it is essential to employ diverse metrics that effectively assess the model’s performance. These metrics play a vital role in quantifying the model’s effectiveness in accurately classifying stress levels. This section presents an overview of the key performance metrics (20) used for evaluating multi-class stress classification models, including precision, recall, accuracy, F1-score, Matthew’s correlation coefficient (MCC), time complexity, and execution time.

### 5.1 Precision

Precision is calculated as the ratio of true positives (TP) to the sum of true positives and false positives (FP). In the context of stress classification models, precision indicates the accuracy of correctly identifying the stress class for each predicted instance. Consequently, the precision can be calculated as

$$\mathrm{Precision}=\frac{\mathrm{TP}}{\mathrm{TP}+\mathrm{FP}}.$$

### 5.2 Recall

Recall, also known as sensitivity, measures the ability of a model to correctly identify positive instances (stress classes) among all instances that belong to the positive class. In stress classification, recall signifies the model’s capability to capture all instances of a particular stress level. Mathematically, recall is the ratio of true positives to the sum of true positives and false negatives (FN). Therefore,

$$\mathrm{Recall}=\frac{\mathrm{TP}}{\mathrm{TP}+\mathrm{FN}}.$$

### 5.3 Accuracy

Accuracy is the ratio of the sum of true positives and true negatives (TN) to the total of all instances (TP, TN, FP, FN). Accuracy is a widely used metric that measures the overall correctness of the model’s predictions across all stress classes. Accuracy can be determined by

$$\mathrm{Accuracy}=\frac{\mathrm{TP}+\mathrm{TN}}{\mathrm{TP}+\mathrm{TN}+\mathrm{FP}+\mathrm{FN}}.$$

### 5.4 F1-score

The F1-score is the harmonic mean of precision and recall, providing a balance between these two metrics. This score is especially useful for imbalanced datasets. The F1-score is computed as 2 times the product of precision and recall divided by their sum, i.e.,

$$F1-\mathrm{score}=\frac{2\times \mathrm{Recall}\times \mathrm{Precision}}{\mathrm{Recall}+\mathrm{Precision}}.$$

### 5.5 Matthew’s correlation coefficient (MCC)

MCC is a correlation coefficient between observed and predicted binary classifications. It provides a balanced measure that can be used even when the classes have disparate sizes. MCC ranges from −1 to 1, with 1 indicating a perfect classification, 0 indicating a random classification, and −1 indicating a completely incorrect classification. The MCC is computed as

$$\mathrm{MCC}=\frac{\mathrm{TP}*\mathrm{TN}-\mathrm{FP}*\mathrm{FN}}{\sqrt{\left(\mathrm{TP}+\mathrm{FP})(\mathrm{TP}+\mathrm{FN})(\mathrm{TN}+\mathrm{FP})(\mathrm{TN}+\mathrm{FN}\right)}}.$$

It is important to note that MCC is a more reliable metric than precision, recall, and F1-score since it avoids dependency on the choice of the positive class and considers the balance among TP, TN, FP, and FN in the confusion matrix.

### 5.6 Time complexity

Time complexity can be seen as the measure of how fast or slow an ML model will perform for the input size (43). It provides an estimate of the number of operations required by a model to solve a problem. Big O notation (tight-bound) is widely used in time complexity analysis. In Big O notation, the time complexity of a model is denoted as:

$$\text{Time Complexity}=O(f\;(n));\forall n\geq n_{0},$$

where f(n) represents a mathematical function that describes the relationship between the input size n and the number of operations performed by an ML model. Lower time complexity indicates faster execution and better scalability.

### 5.7 Execution time

Execution time refers to the duration taken by an ML model to run and complete its execution. The execution time of an ML model can be expressed as:

Execution Time=End Time−Start Time,

where the *Start Time* refers to the time captured immediately before the ML model’s execution begins, and the *End Time* refers to the time captured after the model’s execution is completed.

## 6 Experimental results

In this section, we outline the experimental setup employed in this study. Following that, we will present and discuss the obtained experimental results, including performance metrics and a comparative assessment with existing studies.

### 6.1 Experimental setup

The experimental evaluation was conducted in two phases: simulation-based model development and real-world edge device benchmarking.

In the simulation phase, Google Colab was utilized as the primary platform for training and validating the models. The default Colab environment, equipped with an Intel(R) Xeon(R) CPU (2.20 GHz) and 12 GB RAM, was used throughout. Initially, essential libraries were imported, and the dataset was accessed via a mounted storage system. The input data, originally shaped as (328,257, 34), was reshaped into (328,257, 1, 34) to align with the model requirements. Feature selection methods, including ANOVA and Extra Trees Classifier, were applied to extract the most informative features. Standard preprocessing steps were performed, such as encoding categorical variables, scaling numerical features, and handling missing data. The models (k-NN, Decision Tree, Logistic Regression, and Gaussian Naive Bayes) were instantiated and fine-tuned using grid search. Performance evaluation was carried out using k-fold cross-validation, and metrics including accuracy, precision, recall, F1-score, and MCC were computed. Computational complexity was also assessed through time complexity analysis and execution time measurement.

In the hardware benchmarking phase, we deployed the best-performing model on an NVIDIA Jetson Orin Nano (8 GB) Developer Kit to validate real-world feasibility. The device features an NVIDIA Ampere GPU with 1,024 CUDA cores and 32 Tensor Cores, a 6-core Arm Cortex-A78AE CPU (up to 2.2 GHz), and 8 GB LPDDR5 RAM with 102 GB/s bandwidth.

### 6.2 Feature importance analysis using ANOVA and extra trees

In this study, we focused on the SWELL-KW dataset, which comprises 34 HRV features. During our preliminary analysis, we identified that not all features equally contributed to the classification task, with some having limited relevance in the decision-making process of ML models. To address this, we employed feature selection techniques to identify and retain the most informative features.

**Figure 5:**
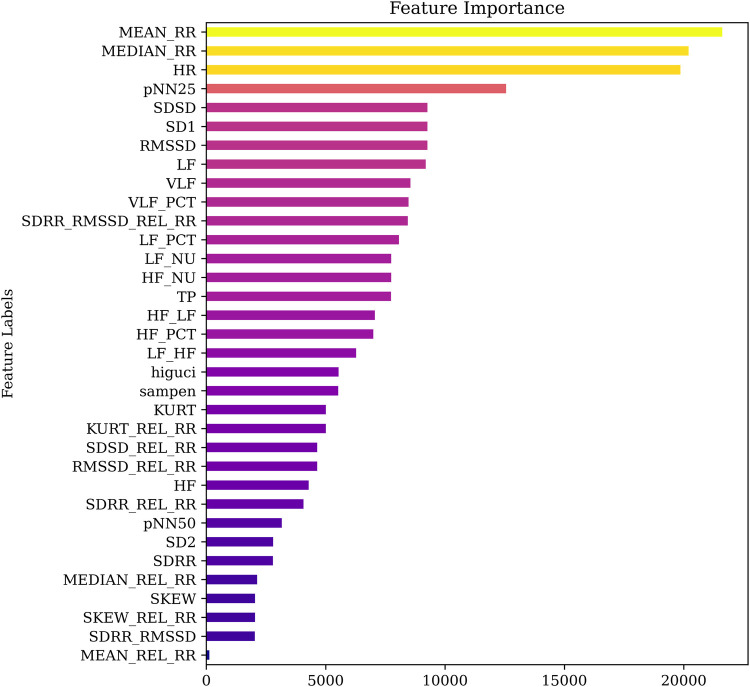
Comparison of feature importance using ANOVA.

As a first step, the ANOVA method was used to rank the features based on their individual F-values. This statistical technique evaluates the variance between and within groups to determine the discriminative power of each feature. The resulting feature importance ranking is shown in Figure 5, where the features are sorted in descending order of significance. For enhanced interpretability, a color gradient was applied, ranging from light (less important) to deep (highly important). From the figure, it is evident that MEAN_RR, MEDIAN_RR, and HR are the top-ranked features, indicating their dominant contribution to stress level classification.

**Figure 6:**
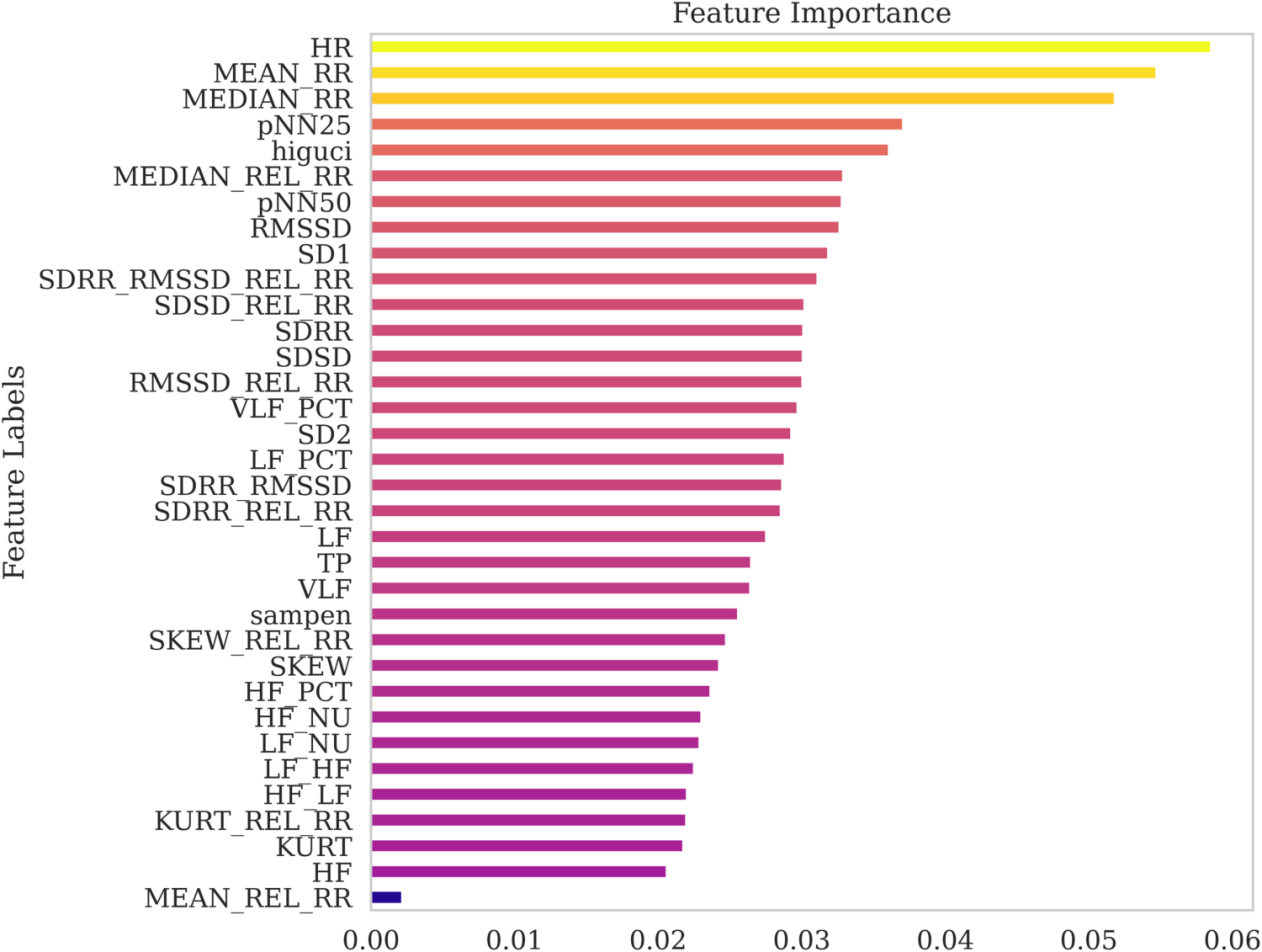
Comparison of feature importance using Extra Tree Classifier.

To validate the robustness of the ANOVA-based rankings, we conducted a comparative feature importance analysis using the Extra Trees ensemble method. The corresponding ranking obtained from this tree-based approach is presented in Figure 6. While some variation was observed in the overall ordering, both methods consistently identified HR, MEAN_RR, and MEDIAN_RR as among the most influential features. This convergence across independent techniques reinforces their significance and supports their selection for lightweight stress detection models.

### 6.3 Performance evaluation

We evaluated the performance of four lightweight ML models, namely k-NN, Decision Tree, Logistic Regression, and Gaussian Naive Bayes, for multilevel stress classification using the SWELL-KW dataset. The models were trained using either the top two or top three features, selected based on the previously described feature importance analysis. To optimize model performance and ensure robust evaluation, hyperparameter tuning was carried out using grid search, and model validation was conducted using 5-fold cross-validation. These steps were taken to reduce the risk of overfitting and to provide an unbiased estimate of the models’ generalization capabilities.

#### 6.3.1 Performance evaluation with top two and three features

To evaluate the effectiveness of lightweight ML models for stress detection using minimal HRV features, we assessed the performance of four classifiers, i.e., k-NN, Decision Tree, Logistic Regression, and Gaussian Naive Bayes using the top two and top three ranked features. The evaluation is based on accuracy, precision, recall, F1 score, and MCC, as shown in Tables 2, 3.

**Table 2:** Model performance using top 3 features.

Model	Accuracy	Precision	Recall	F1 score	MCC
k-NN	0.993	0.993	0.993	0.993	0.988
Decision tree	0.974	0.974	0.974	0.974	0.956
Logistic regression	0.553	0.556	0.553	0.443	0.149
Gaussian Naive Bayes	0.528	0.467	0.527	0.482	0.163

**Table 3:** Model performance using top 2 features.

Model	Accuracy	Precision	Recall	F1 score	MCC
k-NN	0.913	0.913	0.913	0.913	0.853
Decision tree	0.910	0.910	0.910	0.910	0.850
Logistic regression	0.551	0.460	0.551	0.440	0.141
Gaussian Naive Bayes	0.548	0.465	0.548	0.476	0.176

Using the top three features, the k-NN model achieved the highest performance across all metrics, including accuracy, precision, recall, and F1 score of 0.993, and an MCC of 0.988. The Decision Tree model followed closely, with corresponding metric values of 0.974 and an MCC of 0.956. Note that the best hyperparameters for each model were chosen based on the parameters presented in Table 4. With only the top two features, both models continued to show strong performance. The k-NN model attained an accuracy of 0.913 and an MCC of 0.853, while the Decision Tree achieved 0.910 and 0.850, respectively.

**Table 4:** Parameters used in GridSearchCV for tuning with 3 features.

Model	Selected parameters	Best hyperparameter(s)
k-NN	N_neighbors: [3, 5, 7, 9]	N_neighbors: 3
Decision tree	Max_depth: [36, 40, 44, 48]	Max_depth: 44
Logistic regression	Regularization_strength: [5, 10, 15, 20]	Regularization_strength: 15

In contrast, Logistic Regression and Gaussian Naive Bayes models demonstrated substantially lower classification accuracy. For three features, Logistic Regression produced an accuracy of 0.553 and F1 score of 0.443, while Gaussian Naive Bayes recorded an accuracy of 0.528 and F1 score of 0.482. Performance decreased further with only two features, with both models yielding accuracy near 0.55 and lower MCC values.

Tables 5, 6 present the corresponding training times. Gaussian Naive Bayes exhibited the shortest execution time in both cases, requiring only 1.093 s (three features) and 1.392 s (two features). The Decision Tree and k-NN models required moderate execution times ranging from 10 to 15 s. Logistic Regression demonstrated the longest training time with three features (20.197 s), though reduced to 13.691 s with two features.

**Table 5:** Model complexity with 3 features.

Model	Training time (s)
k-NN	15.420
Decision tree	14.027
Logistic regression	20.197
Gaussian Naive Bayes	1.093

**Table 6:** Model complexity with 2 features.

Model	Training time (s)
k-NN	14.699
Decision tree	10.387
Logistic regression	13.691
Gaussian Naive Bayes	1.392

The superior performance of the k-NN and Decision Tree models is attributed to their capacity to handle non-linear relationships in the data. In contrast, the assumptions inherent in Logistic Regression (linear decision boundaries) and Gaussian Naive Bayes (feature normality) are often violated in HRV data, which typically exhibit non-linear and non-Gaussian characteristics. This mismatch results in reduced classification effectiveness for these models.

Adding a third feature improved the classification performance across all models, with the most notable gains observed in k-NN and Decision Tree. These models provided a favorable balance between predictive performance and computational efficiency, making them suitable candidates for stress detection applications using minimal HRV feature sets.

### 6.4 Performance comparison with existing works

The performance of our study on SWELL-KW is compared with other state-of-the-art approaches in Table 7. This table includes information on the type of classification (Binary/Multilevel) denoted by B/M and the number of features represented by No.F. It is worth noting that some existing studies did not report results on precision, recall, and F1 score. Therefore, accuracy was chosen as the metric for a fair comparison since it is commonly available in the existing literature on the SWELL-KW dataset. The results presented in Table 7 clearly demonstrate that our study achieves competitive performance compared to other state-of-the-art approaches. The k-NN model achieves an accuracy of 1.00 (M=5) and 0.99 (M=3), highlighting its effectiveness in classification. Similarly, both the k-NN and Decision Tree models achieve an accuracy of 0.91 (M=2), indicating their strong performance.

**Table 7:** Quantitative comparison of results on SWELL-KW with other state-of-the-art approaches.

Reference	B/M	No.F	Model	Accuracy
Sriramprakash et al. (23)	B	17	SVM	0.92
Sarkar and Etemad (22)	M	34	CNN	0.98
Koldijk et al. (19)	M	34	SVM	0.90
Albaladejo-González et al. (17)	B	34	MLP	0.88
Perangin-Angin and Bachtiar (21)	M	33	ELM/ANN	0.91
Khan et al. (18)	B	8	Extra tree	0.99
Khan et al. (18)	B	14	Extra tree	1.00
Mortensen et al. (20)	M	34	1D-CNN	0.99
Mortensen et al. (20)	M	15	1D-CNN	0.96
This study	M	5	k-NN	1.00
This study	M	3	k-NN	0.99
This study	M	2	k-NN/decision tree	0.91

We also compared the computational complexity of our developed models, k-NN and Decision Tree, with existing works where computational complexity is reported, such as in (20, 21), as shown in Table 8. The results indicate that k-NN and Decision Tree have lower execution times compared to 1D-CNN and ANN. Specifically, the time complexity of k-NN is O(k×n×d) (43), where k, n, and d represent the number of neighbors, instances, and features, respectively. The time complexity of the Decision Tree is O(n×log(n)×d) (43). In contrast, algorithms like ANN and CNN are computationally more expensive.

**Table 8:** Execution time of different algorithms.

Algorithm	Execution time (s)	No. of features
1D-CNN (20)	1733.00	15
ELM/ANN (21)	43.10	33
Decision tree (this study)	14.02	3
k-NN (this study)	15.42	3

Based on these findings, we conclude that k-NN offers a superior combination of higher performance and lower computational complexity.

### 6.5 Overfitting analysis

**Figure 7:**
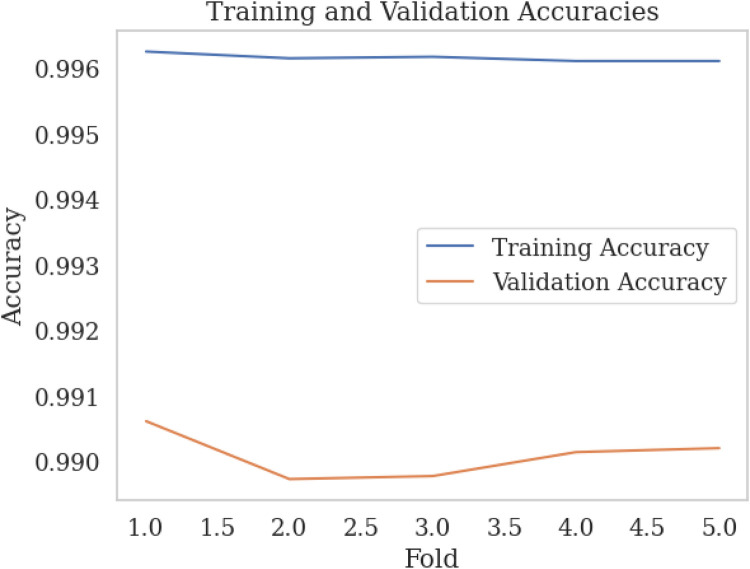
Training vs. validation accuracy over k-fold.

**Figure 8:**
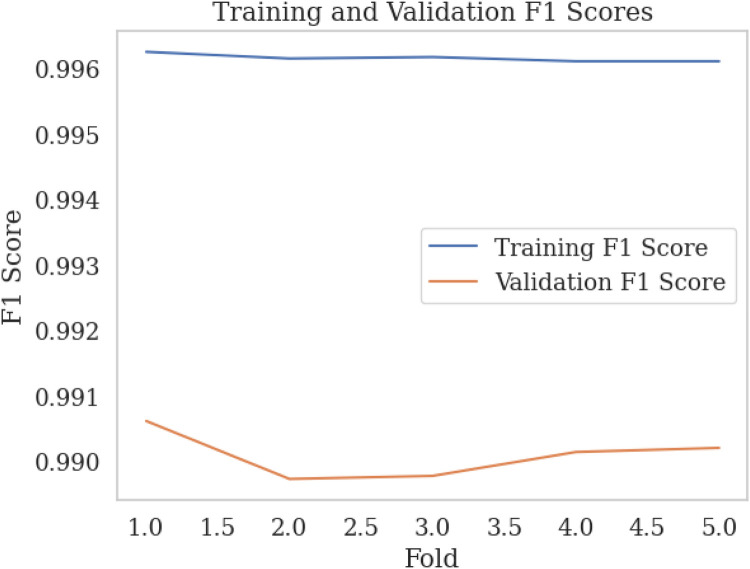
Training vs validation F1 scores over k-fold.

We employed k-fold cross-validation with k=5 to evaluate the performance of our k-NN model with three features from the SWELL-KW dataset, achieving a trade-off between performance and computational complexity. This approach entailed dividing the dataset into k subsets, training the model on k−1 subsets, and assessing its performance on the remaining subset. Figures 7, 8 illustrate the training vs. validation accuracy and training vs. validation F1 score respectively obtained from our experiments. It is worth noting that the validation accuracy closely matched the training accuracy, with a deviation of approximately 0.5%. Similarly, the training vs. validation F1 score exhibited a slight deviation of around 0.5%. These findings indicate that the model did not exhibit overfitting behavior and met the criteria for a well-fitted model.

### 6.6 Interpretability using LIME

The ANOVA testing helped to optimize the HRV feature set to improve the performance and efficiency of the ML models. By eliminating irrelevant and redundant features, ANOVA focused on the most informative ones. The results showed that the k-NN algorithm achieved the highest accuracy using three features: MEAN_RR, MEDIAN_RR, and HR, as identified by the ANOVA method. However, it is important to note that the classification based on these selected features may be perceived as a black box by non-experts. Balancing accuracy and explainability is crucial.

**Figure 9:**
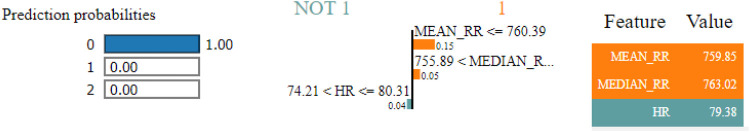
LIME explanation on k−NN classification for top three features and predicting class “0.”

**Figure 10:**
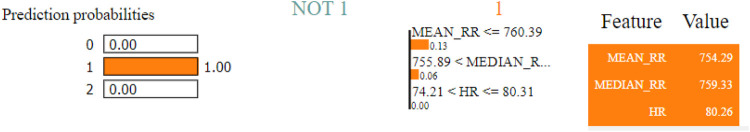
LIME explanation on k−NN classification for top three features and predicting class “1.”

**Figure 11:**
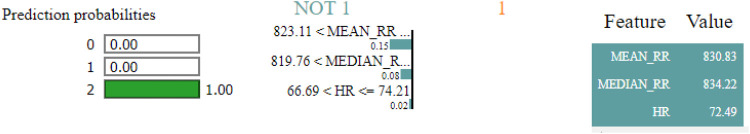
LIME explanation on k−NN classification for top three features and predicting class “2.”

To facilitate a better understanding, Algorithm 1 is utilized to interpret the k-NN classification results using LIME. The experiment takes into account the top three features (MEAN_RR, MEDIAN_RR, and HR) for each predicted class - class “0,” class “1,” and class “2,” which correspond to no stress, interruption, and time pressure, respectively. The outcomes of this experiment are illustrated in Figures 9–11.

**Algorithm 1:** LIME for KNN classification.

1: **Input**: Instance to explain x, KNN model f 2: **Output**: Explanation for the instance 3: **Initialization**: 4: Generate perturbed instances $x^{\prime }$ around x 5: **Initialize** an empty set D for perturbed instances and their labels 6: **for** each perturbed instance $x^{\prime }$ **do** 7: Predict the label $y^{\prime }$ for $x^{\prime }$ using the KNN model f 8: Add $\left(x^{\prime },y^{\prime }\right)$ to D 9: **end for** 10: **Fit** a linear model g to explain f around x 11: **Compute** feature weights using g 12: **return** Feature weights as explanation

The probability of prediction (referred to as “Prediction probabilities” on the left side of the Figures 9–11) in LIME provides crucial information about the trustworthiness and reliability of the model, ranging from 0 to 1. It is utilized to assess the accuracy or incorrectness of a prediction. Furthermore, prediction probabilities influence the weighting of each feature during the generation of explanations. Features that have a significant impact on the prediction probability are given higher importance in the explanation, indicating their relevance in the model’s decision-making process. These probabilities are utilized to identify instances where the model is either highly confident or uncertain about its predictions. This enhances the explainability and reliability of the classification model.

In the LIME explanations, we utilize distinct color codes to underscore the significance of different features. Specifically, *Green* indicates a negative influence or association with the model’s prediction, whereas *Orange* signals a positive one. These color codes greatly aid in interpreting the results for a potential class prediction. More precisely, “Not 1” and “1,” situated in the middle of Figures 9–11, symbolize the negative and positive feature associations for each class, respectively. Further, the terms “Feature” and “Value” shown on the right side of these figures represent the respective influence and measurements of each feature.

As can be observed from Figures 9–11, all three features (MEAN_RR, MEDIAN_RR, and HR) exhibit strong (positive/negative) associations for the prediction of class “1” and class “2.” On the other hand, for class “0,” MEAN_RR and MEDIAN_RR have positive associations, while HR shows a negative association. Overall, all three features play a role in the decision-making process for each class.

It is worth noting that the ANOVA and LIME results do not completely match, and this is due to the different roles they play in the analysis. ANOVA globally ranks the features by looking at the variance across all instances and identifies the top three features as MEAN_RR, MEDIAN_RR, and HR. On the other hand, LIME offers individual explanations for each prediction, showing how these features affect the prediction at a given instance.

### 6.7 Assessment of lightweight ML models for IoT edge deployment

With increasing emphasis on data privacy and compliance with regulations such as the GDPR, on-device ML has become essential in many IoT applications. Local training and inference reduce latency, minimize bandwidth usage, and preserve data privacy. To assess the suitability of the proposed lightweight ML models for IoT edge deployment, experiments were conducted on the NVIDIA Jetson Orin Nano 8 GB module, as described in experimental setup. The k-NN and Decision Tree models were used for stress detection based on the top three HRV features extracted from the SWELL-KW dataset.

On the Jetson platform, the k-NN model achieved an accuracy of 99.26% with a training time of 31.03 s. The Decision Tree model achieved 97.38% accuracy, with a training time of 18.05 s. These results are consistent with the Google Colab based evaluations presented in Section 6.3.1, where both models demonstrated strong classification performance and reasonable training times. The similar results obtained in Google Colab and on the Jetson Nano confirm that the models can be effectively trained on IoT edge platforms for real-time stress detection.

## 7 Limitation

The proposed ML models were validated on an IoT edge device, i.e., the Jetson Orin Nano. We assume that these models can also be trained on the Raspberry Pi 5, as it also supports edge computing. However, these models may not be suitable for deployment on ultra-low-power microcontroller-based platforms, which typically lack sufficient memory and processing capacity. Optimization techniques such as model pruning or quantization may be required for deployment in such environments.

We also acknowledge that the SWELL-KW dataset comprises health data from a limited number of participants, specifically 25 individuals with an average age of 25 years. Variations in HRV across different age groups, ethnicities, and genders can influence the generalizability and reliability of explainable AI methods. Due to the scarcity of HRV datasets encompassing diverse populations, we have not yet been able to verify the robustness of the proposed ML models.

## 8 Conclusion

This study highlights the effectiveness of HRV features for stress detection, achieving high accuracy and computational efficiency ideal for IoT healthcare applications. Using ANOVA for feature selection and grid search for tuning, we developed four machine learning models, i.e., k-NN, Decision Tree, Logistic Regression, and Gaussian Naive Bayes. The k-NN model stood out with a 99.3% accuracy using only three features, outperforming others. Incorporating LIME enhanced interpretability, reinforcing transparency in healthcare AI. While advanced machine learning models hold potential, our findings confirm that traditional classifiers like k-NN and Decision Trees offer an optimal balance of accuracy, speed, and interpretability on resource-limited devices. Given the task-specific requirements, these simpler models provide reliable, efficient, and explainable solutions for IoT-based stress detection systems. Our future work will focus on improving robustness and generalizability by incorporating datasets with diverse age groups.

## Data Availability

The original contributions presented in the study are included in the article/Supplementary Material, further inquiries can be directed to the corresponding author/s.
